# Breath-to-breath respiratory mechanics variation: how much variation should we expect?

**DOI:** 10.1186/cc14340

**Published:** 2015-03-16

**Authors:** KT Kim, YS Chiew, C Pretty, GM Shaw, T Desaive, JG Chase

**Affiliations:** 1 University of Canterbury, Christchurch, New Zealand; 2 Christchurch Hospital, Christchurch, New Zealand; 3 University of Liege, Belgium

## Introduction

Model-based respiratory mechanics can be used to guide mechanical ventilation therapy. However, identified mechanical properties vary breath to breath, leading to potential treatment errors when using model-based care that requires accuracy. This study investigates and quantifies this variability to improve its application in guiding clinical interventions.

## Methods

Retrospective data from 12 acute respiratory distress syndrome (ARDS) patients were used [1]. Each patient was sedated to prevent spontaneous breathing effort, and ventilated using the volume control mode with a square flow profile. Varied PEEP levels were maintained for 30 minutes before 1 minute of data were collected for analysis. This dataset provides a wide range of respiratory mechanics values, and the clinical protocol detail is in [1]. A clinically proven, single compartment model respiratory system elastance (Ers) is identified from data for every breathing cycle at each PEEP level using a linear regression method. The dynamic elastance (Edynamic = (peak airway pressure - positive end-expiratory pressure) / tidal volume) of the corresponding breathing cycle is calculated for comparison.

## Results

The coefficient of variation (CV) of identified Ers across all patients was low (<0.005), as expected, as the 30-minute period allows time-dependent alveolar recruitment to fully occur and stabilise. However, even with substantial stabilisation periods, there remains a difference between the minimum and maximum estimated Ers within each 1-minute period of analysed data. The differences were median 2.8% (interquartile range (IQR): 1.5 to 4.6%, 90% range (90R): 0.8 to 13.4) for Ers and 3.6% (IQR: 2.3 to 5.2, 90R: 0.9 to 10.8) for Edynamic, and in extreme cases, can be up to 16%.

## Conclusion

This study quantified the variability (over short periods) of identified and estimated respiratory mechanics properties used to (potentially) guide ventilation care in sedated patients. It is also important to note that this minimum level of variability occurs even when stabilisation is achieved. Thus, clinically, if this information was to be used to guide ventilation in real time, such as titrating PEEP to minimal elastance, larger errors, at least up to 15% variation in Ers, could be expected, which could well affect care. Such levels thus also begin to define the minimum levels of change necessary to be larger than natural variation.
